# Evaluation of Pre-analytical Errors in the Histopathology Laboratory at a Tertiary Care Hospital in Kenya

**DOI:** 10.7759/cureus.116635

**Published:** 2026-09-21

**Authors:** Josephine N Rioki, Rency Lel, Allan T Kirrima, Mwangi Joseph

**Affiliations:** 1 Department of Medical Laboratory Sciences, University of Nairobi, Nairobi, KEN; 2 Centre for Virus Research, Kenya Medical Research Institute, Nairobi, KEN; 3 Department of Research, Kenya Medical Research Institute Graduate School, Nairobi, KEN

**Keywords:** histopathology, laboratory informatics, pre-analytical phase, quality indicators, rejection criteria

## Abstract

Background: Pre-analytical errors comprise inappropriate performance and procedures executed before sample analysis. These nonconformities compromise report quality, consequently impeding optimal patient management and clinical outcomes. Awareness of these errors within histopathology laboratories is crucial for maintaining diagnostic integrity.

Objective: This study aimed to characterize the frequency, nature, and distribution of documented demographic, accessioning, and specimen-rejection errors recorded in the Kenyatta National Hospital (KNH) histology laboratory registers between December 2017 and December 2019.

Methodology: A retrospective cross-sectional study was conducted reviewing data from the general laboratory register and the sample rejection register recorded between December 2017 and December 2019. Assessment focused on two major domains: demographic/accessioning errors and sample rejection criteria. Analysis was performed using IBM SPSS Statistics software, version 20 (IBM Corporation, Armonk, NY, USA).

Results: A total of 757 missing patient information records were recorded during the study period. Missing patient age was the most prevalent demographic error among the missing data events, accounting for 52.2% (24/46) in December 2017, 58.6% (286/488) in 2018, 57.4% (128/223) in 2019, and overall 57.9% (438/757). Within the sample rejection category, a missing request form was the most frequently specified reason for rejection (16.0%; 8/50 in December 2017, 40.0%; 2/5 in 2018, and 60.0%; 3/5 in 2019) and overall, 21.7% (13/60). Crucially, in December 2017, 66.0% (33/50) of the samples were rejected without documentation of the specific reason for rejection.

Conclusion: Requisition forms were systematically underfilled, with missing patient age representing the predominant demographic discrepancy. Missing requisition forms reported with the highest frequency in this study were strongly associated with sample rejection. Furthermore, a severe lack of structured documentation regarding the reasons for specimen rejection was identified.

## Introduction

Laboratory medicine is a complex field comprising the pre-analytical, analytical, and post-analytical phases of the diagnostic process. The pre-analytical phase comprises all steps ranging from test ordering, filling of laboratory requisition forms, patient identification, specimen collection, and transport to accessioning and data entry within the laboratory [1]. Lapses in these steps are sources of pre-analytical errors [2]. Historically, quality assurance programs have focused heavily on the analytical phase. While technological advancements have significantly reduced errors within the analytical phase, the pre-analytical phase remains the most vulnerable and error-prone segment of the diagnostic process, accounting for up to 70% of laboratory errors [3]. The clinical risk associated with these errors is profound. In histopathology, the implications of pre-analytical errors are uniquely irreversible.

Histopathology reports are regarded as the gold standard for tissue-based diagnosis of many diseases, particularly cancers. In tissue diagnosis, the risks and consequences of the preparatory stage are exceptionally critical. Unlike a blood sample, which can often be recollected with minimal patient harm, a histological specimen is often irreplaceable. An error in fixation, labeling, or clinical documentation during this phase can lead to irreversible diagnostic failure, often requiring additional invasive procedures that expose the patient to further risk [4, 5]. The consequences of diagnostic failure are starkly illustrated by a previous report, where characteristic MRI features and multiple biopsies failed to yield a definitive diagnosis, with the correct diagnosis only established at histopathological examination [6]. This case underscores that even when appropriate investigations are performed, diagnostic failure can occur, reinforcing the critical importance of pre-analytical quality in histopathology.

Unlike clinical chemistry or hematology, where a sample can often be recollected easily, histological specimens frequently consist of whole organs, deep biopsies, or unique surgical tissues that cannot be re-obtained without secondary invasive procedures. Consequently, quality assessment across pre-analytical phases, including sample collection in the operating theater, transit to the dissecting room, and subsequent data accessioning, is a clinical requirement [4]. Common histopathological pre-analytical errors include inappropriate transcription, missing demographic profiles, incorrect labeling, and inadequate fixative volume or type [7].

At Kenyatta National Hospital (KNH), a leading tertiary care facility in Nairobi, Kenya, a preliminary evaluation by Kimengech et al. highlighted that pre-analytical errors compromised service delivery with a substantial baseline frequency of 42.8% [8]. Another study by Osimbo et al. 2018 in the same facility found that “patient location and clinical information/diagnosis were completed in 55.9% and 55.5% of the forms, respectively” [9].

Despite this awareness, comprehensive audits evaluating the patterns of individual demographic discrepancies and structural data documentation remain scarce. This study was designed to characterize the nature, trends, and specific frequencies of pre-analytical errors at the KNH histopathology laboratory over a three-year period to identify system vulnerabilities and guide targeted corrective interventions.

## Materials and methods

Study design and setting

This study utilized a retrospective cross-sectional design. The investigation was situated at the KNH Histology Laboratory in Nairobi, Kenya. This tertiary facility processes diverse histopathological specimens acquired from inpatient wards, outpatient clinics, surgical theaters, and the KNH mortuary for postmortem evaluation.

Study population and data sampling

The study population encompassed all archived administrative and operational data fields documented within the general laboratory register and the official specimen rejection register. The census sampling technique was deployed to capture all recorded nonconforming activities logged between December 2017 and December 2019.

Inclusion and exclusion criteria

All data points reflecting data entry omissions, specimen label discrepancies, accessioning errors, and documented rejections within the specified period (December 2017 to December 2019) were included. Laboratory entries falling outside this window or containing missing records for consecutive historical blocks were excluded.

Data collection protocol

Data extraction was facilitated via a standardized data collection sheet. Data fields captured fell into two distinct domains.

Demographic and Accessioning Variables

This included completion status of fields including patient age, sex, hospital identification number, requesting clinic/ward unit, and specified tissue/specimen type.

Rejection Variables

This included incidences of missing labels, missing request forms, missing samples, non-fixed tissues, and instances where sample rejection lacked recorded reasons.

Definition of the audited categories operationally

A demographic or accessioning error was defined as a required field recorded as blank or incomplete in the register, including patient age, sex, and hospital number, originating unit or clinic, and specimen type. A documented specimen rejection was defined as an entry in the specimen rejection register indicating that the specimen was not accepted for processing. Rejection categories included missing requisition form, missing specimen, and missing or unlabeled specimen container, inadequate or absent fixation, and rejection without a recorded reason.

The data were extracted using a standardized data-collection sheet from the general laboratory register and specimen rejection register. The registers captured recognized and documented events only; therefore, the audit did not measure unrecognized errors or errors corrected before documentation.

To maintain strict patient confidentiality, codes were assigned to records instead of utilizing direct patient identifiers. Collected data was maintained on a password-encrypted hardware system.

Data analysis

Statistical analysis was done using IBM SPSS Statistics software, version 20 (IBM Corporation, Armonk, NY, USA). Categorical data were converted into descriptive statistics, specifically frequencies and percentages. Annual variations across December 2017, 2018, and 2019 were tabulated systematically to track operational trajectories.

## Results

During the evaluation period spanning December 2017 to December 2019, a total of 757 missing data-events were documented in the laboratory reflecting instances of missing or incomplete patient information . Concurrently, the laboratory logged 60 definitive sample rejections.

Demographic and accessioning errors

The distribution of demographic data omissions was assessed over the study period(Two years, one month). Missing age consistently emerged as the most (57.9%;n=438) frequent operational error across the study period. The distribution of these errors is as shown (Table 1).

**Table 1:** Annual and cumulative distribution of demographic and accessioning errors

Pre-analytical Error Category	December 2017 Frequency (%)	January-December 2018 Frequency (%)	January-December 2019 Frequency (%)	Total Frequency (%)
Missing patient age	24 (52.2%)	286 (58.6%)	128 (57.4%)	438 (57.9%)
Missing hospital number	12 (26.1%)	72 (14.8%)	38 (17.0%)	122 (16.1%)
Missing origin unit/clinic	3 (6.5%)	57 (11.7%)	32 (14.3%)	92 (12.2%)
Missing patient sex	5 (10.9%)	43 (8.8%)	9 (4.0%)	57 (7.5%)
Missing type of specimen	2 (4.3%)	30 (6.1%)	16 (7.2%)	48 (6.3%)
Total errors	46 (100%)	488 (100%)	223 (100%)	757 (100%)

Specimen rejection trends and parameters

A total of 60 specimens were rejected over the course of the study period. In December 2017(one month) alone, 50 samples were rejected. A critical documentation deficit was observed, as 66.0% (n=33) of those rejections occurred without any recorded justification. In contrast, the documentation rate reached 100% by 2019, with missing requisition forms constituting the leading reason for rejection (Table 2).

**Table 2:** Chronological profile of reasons for sample rejection (2017–2019)

Identified Reason for Sample Rejection	December 2017 Frequency (%)	January-December 2018 Frequency (%)	January-December 2019 Frequency (%)	Total (Cumulative) Frequency (%)
Reason for rejection not documented	33 (66%)	0 (0%)	0 (0%)	33 (55%)
Missing requisition form	8 (16%)	2 (40%)	3 (60%)	13 (21.7%)
Missing specimen sample	8 (16%)	1 (20%)	2 (40%)	11 (18.3%)
Missing/Unlabeled specimen container	1 (2%)	1 (20%)	0 (0%)	2 (3.3%)
Inadequate/Absent tissue fixation	0 (0%)	1 (20%)	0 (0%)	1 (1.7%)
Total annual rejections	50 (100%)	5 (100%)	5 (100%)	60 (100%)

## Discussion

The occurrence of pre-analytical errors in histopathology is a major driver of diagnostic delays and compromised diagnostic accuracy. This retrospective study demonstrates a high incidence of incomplete clinical requests and highlights critical gaps in structural documentation within the KNH histology laboratory. 

Dynamics of demographic and accessioning fault lines

Omissions within demographic parameters, specifically missing patient age (57.9% overall) and missing patient sex (7.5% overall), represent a substantial systemic challenge. This trend closely mirrors the findings of others who noted an equivalent 50% [10] baseline omission rate for age and sex details on clinical lab requests. A study by Nya Kemkeu et al. found at least one nonconformity in 61.54% of specimens. In contrast, a significantly lower age omission frequency of 13% was reported by other researchers [11]. This variance likely stems from differences in study design; Baqui et al. conducted a prospective tracking study over a tighter observation frame, which may have introduced a Hawthorne effect among clinical staff [12].

Demographic data is not merely administrative; it serves an important clinical role. In histopathology, age and sex provide essential epidemiological context necessary for differential diagnosis. For instance, encountering a small round blue cell tumor yields entirely different diagnostic probabilities depending on whether the patient is a pediatric or a geriatric individual. Furthermore, the absence of this information impedes retrospective data correlation and limits the validity of institutional cancer registries.

Accessioning errors, such as missing hospital identification numbers (16.1%) and missing source units (12.2%), were less frequent but remain clinically significant. Our finding of a 16.1% missing hospital number rate is lower than the 44% noted by previous research [12]. Because our study was retrospective, it is highly probable that clinicians retroactively corrected many of these errors before formal registration to prevent administrative rejection.

Omissions regarding the requesting clinic or ward (12.2%) align with the 10% rate observed by Baqui et al. [12]. Clinicians frequently overlook these parameters due to poor requisition form design or a perception that the hospital number alone is sufficient. However, omitting the originating unit severely delays the dispatch of critical tissue reports and complicates patient tracking.

Specimen rejection profiles and administrative deficits

The analysis of the sample rejection register revealed that missing requisition forms accounted for 21.7% of total rejections over the study period. A missing requisition form introduces substantial clinical risk, as it strips the laboratory of vital information regarding tissue origin, structural topography, and anatomical landmarks.

Interestingly, instances where a requisition form was delivered without the matching specimen accounted for 18.3% of cumulative rejections. This represents a distinct problem compared to the 0% rate reported by Rao et al. [4]. This type of error typically occurs during specimen transit, often as a result of support staff inadvertently misdirecting tissue containers to the clinical chemistry or hematology laboratories instead of the histopathology laboratory.

Only one sample (1.7%) was rejected due to improper fixation, which aligns with the low rate (0.17%) documented by Rao et al. [4]. This low error rate is likely because the KNH histology laboratory prepares standard 10% neutral buffered formalin in-house and distributes it directly to the surgical theaters.

The most alarming finding in this study was that 55.0% of cumulative sample rejections (33 instances in December 2017) lacked any documented justification. This contrasts sharply with the 0% undocumented rate reported by Rao et al. [4]. This deficit points to a lack of structured quality management and data accountability during that period. Other studies have reported specimen identification and delivery errors as significant sources of preanalytical errors in pathology laboratories [13].

Without a clear, recorded reason for rejecting a specimen, it is impossible to implement targeted quality improvements. Encouragingly, the data show a positive trend: undocumented rejections dropped from 66.0% in December 2017 (one month) to 0% in 2018 and 2019. This improvement may be an indication that the laboratory successfully adopted more rigorous documentation protocols over the study period.

Based on the findings of this study, several recommendations can be implemented to minimize pre-analytical error rates in histopathology laboratories and, consequently, improve patient care outcomes. First, laboratory-clinician training should be strengthened for clinical residents, medical officers, and surgical theater teams, with emphasis on the clinical importance of accurately and completely filling out histopathology requisition forms. Second, clear protocols and checklist-based training should be developed for hospital support staff and specimen messengers to ensure that specimens and their corresponding requisition forms are properly labelled, verified, and matched before delivery to the laboratory. Finally, laboratory registers should be updated to include specific fields for documenting pre-analytical errors, with pathologists and laboratory technologists required to record the explicit reason for rejecting each specimen. These measures would promote accountability, improve communication between clinical and laboratory teams, and support continuous quality improvement in histopathology services.

Limitations of the study

Its retrospective design relied exclusively on general laboratory and specimen rejection registers; therefore, only recognized and documented pre-analytical errors could be identified, while undocumented or unrecognized errors may have been missed. Although pragmatic, the use of register-based data may not fully capture the extent of pre-analytical errors. The study was also conducted at a single tertiary referral hospital, limiting generalizability to other Kenyan healthcare settings with different specimen volumes, staffing, referral systems, laboratory information systems, and quality management practices. Data from 2017-2019 were selected to establish a stable pre-pandemic baseline, avoiding COVID-19-related disruptions and enabling future comparisons. However, observation periods were unequal, as December 2017 represented only one month, whereas 2018 and 2019 covered full years. Therefore, differences should be interpreted descriptively rather than as annual trends. The decline in undocumented sample rejections cannot be attributed to a specific cause. Future prospective post-pandemic studies are recommended to assess sustained improvements and emerging trends.

## Conclusions

This retrospective register audit identified frequent documented omissions in histopathology requisition data and recurrent documented specimen rejection events at the KNH histology laboratory. Missing patient age was the most frequently recorded demographic omission, while missing requisition forms were the most common categorized reason for rejection. Because the observation periods were unequal and the study was retrospective, the findings describe documented patterns and identify areas for quality improvement but do not establish temporal causality or clinical consequences.
